# Feeding back of individual genetic results in Botswana: mapping opportunities and challenges

**DOI:** 10.1186/s12910-023-00912-1

**Published:** 2023-06-03

**Authors:** Mary Kasule, Mogomotsi Matshaba, Ambroise Wonkam, Jantina de Vries

**Affiliations:** 1 Botswana-Baylor Children’s Clinical Centre of Excellence, Gaborone, Botswana; 2 Baylor College of Medicine, Houston, TX USA; 3 Deputy Dean’s Office, Faculty of Health Sciences and Groote Schuur, Cape Town, South Africa; 4 Department of Medicine and NeuroScience Institute , The Ethics Lab, Cape Town, South Africa

**Keywords:** Opportunities, Challenges, Regulatory oversight, Actionability, Resources, Cost

## Abstract

**Purpose:**

We explored the views of Botswana stakeholders involved in developing, implementing and applying ethical standards for return of individual study results from genomic research. This allowed for mapping opportunities and challenges regarding actionability requirements that determine whether individual genomic research results should be fed back.

**Methods:**

Using in-depth interviews, this study explored the views of sixteen (16) stakeholders about the extent, nature and timing of feedback of individual genomic research findings, including incidental findings that arise in the context of African genomics research. Coded data was analyzed through an iterative process of analytic induction to document and interpret themes.

**Results:**

Overall, respondents were of the view that feedback of actionable individual genomic results was an important outcome that could benefit participants. However, a number of themes surfaced that pointed to opportunities and challenges that exist in Botswana that could help in planning for feeding back of individual genomic results that were mapped. Some of the opportunities cited by the respondents included the existence of good governance; democracy and humanitarianism; universal healthcare system; national commitment to science; research and innovation to transform Botswana into a knowledge-based economy; and applicable standard of care which could promote actionability. On the other hand, contextual issues like the requirement for validation of genomic research results in accredited laboratories, high cost of validation of genomic results, and linkage to care, as well as lack of experts like genomic scientists and counselors were considered as challenges for return of individual results.

**Conclusion:**

We propose that decisions whether and which genomic results to return take into consideration contextual opportunities and challenges for actionability for return of results in a research setting. This is likely to avoid or minimize ethical issues of justice, equity and harm regarding actionability decisions.

**Supplementary Information:**

The online version contains supplementary material available at 10.1186/s12910-023-00912-1.

## Introduction

The quantity of genomic data generated about research participants in African populations by initiatives such as the Human, Health and Heredity in Africa (H3Africa) is rapidly increasing [1]. A deeper understanding of this data could arguably benefit research participants when translated into health care interventions [2]. This could translate into a strong argument to afford research participants the opportunity to receive at least some of their individual findings. Yet few studies have analyzed what genomic research professionals in Botswana and Africa more broadly think about this issue and what they are doing to address it [3]. Therefore, questions about whether and which results ought to be fed back in genomics research have become an area of growing concern on the African continent. The emerging consensus from international [4, 5] as well as H3Africa guidelines [3] is that at least some findings in genetic research must be returned to individual donors if they wish, especially those that satisfy the standard actionability. “Actionability” pertains to the presence of an intervention to prevent, treat or improve the condition predicted or signalled by an incidental finding. However, this actionability only exists if appropriate resources are available [6]. Yet there are unique considerations around actionability when genomics research takes place in lower or middle-income countries where healthcare systems may be severely under-resourced and where research participants are often unable to afford private healthcare. In that setting, what results are to count as actionable is not always clear.

In a publication by Ortiz-Osorno, [6], the authors developed the ‘Actionability at the Participant Research Setting Level’ (APRSL) model to describe the practical fluidity of the actionability requirements that determine whether individual genetic research results should be fed back. They argue that actionability varies from setting to setting, depending on the availability of resources. Therefore the setting should be the “driving force” in determining whether and what individual genetic results will be returned especially in the case of multi-site studies, where there may be wide variability in available medical resources as well as cultural diversity. This variation could be due to: (a) the availability of required resources; (b) the actual costs of those resources; (c) financial support available to participants; (d) the degree of referral programs and linkages to care; and (e) the level of expertise required and available in each study setting. These variations would be dependent on the opportunities and challenges for availability of necessary resources in a setting. Whilst a valuable contribution to thinking about the return of individual genetic research results in international genomics research, the application of the APRSL model raises a number of questions in the African context. For instance, in African countries there may be considerable variation in what is available in urban areas versus rural areas in terms of health financing, cultures, health literacy, social-economic status and levels of education. To explore the usefulness of the APRSL approach, we critically interrogated the Botswana research setting where genomic research is still in its infancy in terms of technological advancement.

## Methods

### Setting

Botswana is located at the center of Southern Africa with significant mineral wealth, good governance, and a relatively small population of slightly above 2 million people. This has elevated the country to an upper middle-income country with the aim of becoming a high-income country by 2036 [7] and allowed the country to make strides in universal healthcare access for much of its population [8]. The total Health Expenditure as a percentage of the GDP is 5.4% [9]. Public sector healthcare services are almost free for citizens whilst non-citizens pay a subsidized fee. Patients pay a nominal cost recovery system through a fee of approximately 0,5 US dollars at the point of service, with the exemption of vulnerable populations (children, pregnant mothers, the aged and specified communicable diseases). The country also has private insurance in the form of medical aid schemes but only people in the higher-income brackets have access to this. Botswana governance is characterised by a consultative culture of dialogue and humanitarianism, and a concept of “Botho” [10] which promotes solidarity and reciprocity. Most people in Botswana live in semi-urban villages (43.0%) that are close to big cities while some live in rural villages (36.1%) or in cities and towns (20.9%) [11]. Of note, large biomedical research institutions are located in the cities and towns, while the majority of the potential research participants come from semi-urban and rural villages. Due to a falling income from diamonds, Botswana is committed to transforming its economy from a resource economy into a knowledge-based economy through research, science, technology and innovation. This will be achieved through the Ministry of Tertiary Education, Research, Science and Technology [12] and institutions like the Botswana Innovation Hub (BIH) [13], Botswana International University of Science and Technology [14] and Botswana Institute for Technology Research and Innovation (BITRI) [15]. However, Botswana still has challenges like lack of sufficient manpower to utilize the resources available and a small population for consumption of products invested in. This requires regional and global collaboration.

Despite the above successes, high rates of income inequality have led to an estimated 16.3% of the population living below the national poverty line [16]. Furthermore, unemployment is estimated to be 18.1%. Poverty is more prevalent in rural areas, among female-headed households and the youth and lowly-skilled people [17]. This situation is likely to have a bearing on feedback of actionable results as income inequality affects affordability of treatment and prevention. Furthermore, levels of education and languages used to communicate information in genomics research can also impact on feedback of actionable results. Botswana’s literacy rate stood at 90% in 2014, with greater literacy in towns and cities compared to rural areas [18]. Regarding languages spoken in Botswana, an estimated 70% of the population is ethno-linguistically homogenous and speaks Setswana which is the national language, although English is considered as the official language. The other 30% of the population speaks 28 other languages [19]. Therefore, education levels and languages used to communicate in research could have a huge bearing on the readability and comprehension of information provided to potential research participants through the informed consent process. Low education levels could translate into low health literacy as observed elsewhere [20] which would make the feedback of complex genetic results difficult to comprehend, impacting on participants’ feedback decision making.

An established research governance and oversight system for research involving human subjects has existed since the 1980s. For example a mandatory requirement for a research permit before commencement of any research in the country is in place; country-wide Institutional Review Boards (IRBs) at academic and institutional level as well as Community Advisory Boards (CABs) mostly linked to IRBs have also have been established. Routine training of IRB and CAB members both locally and internationally is also a requirement by the research regulations [21, 22]. However the capacity of IRB members to review genomic research has not been assessed. With regard to policies, Botswana has a national health policy [23] that emphasizes research and development, health financing and health technologies among other things. National guidelines exist in form of Standard Operating procedures (SOPs), that guide the conduct of genomic research procedures, section:7.2 (iv). Although in terms of feedback of findings the SOPs have a provision which states that *“participants are informed during the consenting process that the researchers will endeavour to provide information about the outcome of the research, and when it is not intended to provide feedback”* [24], they lack detail on the conditions that determine the return of genetic results. Legally Botswana constitution provides for fundamental rights and freedoms of every individual [25] and a Data Protection Act No. 32 of 2018 (Sects. 23–26) [26]. All these oversight guidelines point to opportunities for feedback of individual results in Botswana.

Our study was hosted by Botswana-Baylor Children’s Clinical Centre of Excellence which led a multi-county genomics Collaborative African Genomics Network (CAfGEN), an H3Africa genomic project conducted in Botswana, Eswathini and Uganda. The CAfGEN study aimed to identify host factors that are important to the progression of HIV and HIV-TB infection among children [27]. We explored the perspectives of stakeholders in Botswana involved in developing, implementing and applying ethical standards and policies for return of individual results on feeding back individual genetic research results, some of the responses from the in-depth interviews enabled mapping existing opportunities and challenges in Botswana for developing guidelines or standard operating procedures for feedback of individual genetic results best practice.

### Study population

Respondents included stakeholders from academia, research institutions and government ministries in Gaborone, the capital city of Botswana that are involved in developing, implementing and applying ethical standards and policies for biomedical and Sociobehavioural research involving human subjects, including genomics research. These included healthcare providers, university lecturers, ethics committee members, community advisory board members, researchers and medical genetics professionals who had been involved in the conduct of or and regulation of research involving human subjects for at least two years.

### Sampling, data collection and analysis

Twenty seven (27) potential respondents were invited to participate in the study and sixteen (16) agreed to participate while nine (9) did not respond to our invitation and 2 could not honor their appointments. Data was collected by (MK) assisted by a research assistant (RW) both of whom are trained and experienced interviewers familiar with qualitative research methods. A qualitative methodology using an in-depth interview (IDIs) questionnaire developed by the research team was used for data collection [28]. All participants were sent an invitation letter introducing them to the aims and objectives of the study as well as an Information Sheet. Each participant was also given a brief background of the researcher and the study prior to commencement of the interview. The interviews were conducted in English at the respondents’ workplaces, and lasted approximately 45 to 60 min. All interviews were audio-recorded with permission from the respondents and were later transcribed verbatim. Interviews were conducted until saturation was reached [29], which we established through interim data analysis. No personal identifiers were collected, no master list was maintained that could link transcripts to respondents and audio recordings were assigned study identification codes. Established procedures for qualitative research methods were followed to ensure rigour and trustworthiness of data collection, coding and analysis procedures [30, 31]. Briefly, transcripts were first checked for accuracy and familiarization with data. Thematic analysis was conducted by two of the authors (MK and JDV) and the interview texts were then analysed for content in line with the study aims. Guided by the objectives of the study, we initially open-coded selected transcripts to search for relevant concepts and a hierarchical coding scheme was used to identify the main study themes and sub-themes to generate a codebook. All transcripts were uploaded to and analysed in NVivo qualitative Version 12 (QSR) International Pty Ltd, 2012) software to aid in indexing, searching and retrieving sections of data. In-depth analysis of the coded data was conducted through an iterative process of analytic induction to document and interpret themes and patterns.

## Results

### Demographic characteristics

Table 1 shows that the sixteen (16) respondents were those who had either conducted or regulated human research for at least the last two years previously some with responsibilities in genomics research, patient care, policy development, ethics regulation and community engagement in Botswana. They responded to our in-depth interviews between July 2019 and June 2020. Their ages ranged between 40 and 65 years and majorities were male. All respondents were highly trained professionals with specializations in various biomedical and social behavioral fields. They also held various positions and performed other research related responsibilities. All 16 respondents had basic training in genetics which gave them good background knowledge of genetics and heredity. Unfortunately, only a few had specialized training in genomics at graduate or postgraduate level and had participated in genomics research.

**Table 1:** Respondents’ Social- Demographic characteristics

Characteristic	Item	Count
Gender	Male	10
	Female	6
Age	41–50	9
	51–59	2
	> 60	5
Highest level of education	Graduate	4
	Masters	1
	PhD	5
	MD	6
Other Specializations	Epidemiology	3
	Biostatistics	3
	Pediatrics	1
	HIV research	5
	Health Financing	2
	Protection of Human Subjects certificate	14
	Health Policy	2
	Bioethics	1
Research experience	5–10 years	1
	11–15 years	2
	16–20 years	1
	> 20 years	5
Position	Medical Doctor	6
	Laboratory Scientist	3
	Lecturer	3
	Nurse	3
	Policy	3
	Study Coordinator	3
Research responsibilities	IRB member	6
	Principal Investigator (Biomedical)	5
	Principal Investigator (Social Sciences)	2
	Research Manager	4
	Community Advisory Board member	2
	Ethics Regulator	3
	Health Economics	2
Genomics	Background Knowledge & Knowledge about heredity	16
	Genomics Research	8

Overall, all 16 respondents supported feedback of actionable individual genetic results. They were of the view that this practice was an important outcome that could benefit participants. However, our results revealed a number of contextual issues that we categorized as themes under either opportunities or challenges. Our Category of Opportunities included: Botswana’s democratic governance and the free universal health care system. Other opportunities noted were the national commitment to science, research and innovation; and the mandatory provision of applicable standard of care. Our Category of Challenges included: the process and cost of feeding back results validated in an accredited laboratory; linkage to care; and the non-availability of experts in genomics research.

Our respondents were not so much concerned about whether or not participants could afford prevention and/ or follow-up treatment for validated conditions discovered from genomics research. This was attributed to the comparatively strong Health Care system which provides Universal Health Care supported by Botswana’s democratic governance and economic management which ensure that the values and principles of stewardship, transparency, participation, fairness, accountability and following the rule of law are adhered to. With this type of system, most respondents felt that despite the high costs of validation of results, follow-up treatment, and referrals, these would likely, partly or fully, be borne by government or private medical insurance.

Therefore, overall all respondents felt that feedback of actionable individual genetic results in Botswana is possible because of the universal health care system as captured by this sample response below:

IDI 01 *“Our health system is a free kind of health system, or health for all in Botswana whereby everyone has got access. … Where something can be done the fact that our health system is– like this. We always pride ourselves, saying that health care is available to everyone; the facilities are within 5 kilometre radius for every settlement. You see, and you know that the referral is also there. The health care system is there with a wide coverage”.*

Some respondents however cautioned that although health services are free for all Botswana nationals, there are some inequity contextual challenges like low levels of education, low genomic literacy and poverty among the majority of research participants that can impact on actionability. These factors can affect comprehension of information fedback to participants, access to necessary genomic information as well as the cost of follow-up and care since the majority of participants may not be able to afford out-of-pocket services and lack of medical insurances. One respondent however noted that there could be solutions to these factors to enable return of actionable results. For example, empowering participants and communities through simple education that can be provided at the regular public meetings or community councils of Botswana villages referred to as “kgotla” meetings presided over by the village chief or headman. Here community issues are discussed to articulate people’s needs through dialogue. Village Development Committees (VDCs) are also available where such education can be provided mainly to promote transparency and trust as well as minimize stigma attached to some genetic diseases. This was expressed by one respondent as follows:

*P04: “Like majority of people I take care of at the hospital are poor people as they do not have private insurance! Many of them understand when/if you take time to explain things. They require a kind of education, so they can appreciate and understand what you are saying to them”.*

Some respondents felt that due to the high costs of treatment of most genomic conditions, results for such conditions should not be fedback because government might not afford. However, some were of the opinion that even such results should be fed back based on the existing cultural concept of “Botho” a Botswana concept of reciprocity and solidarity. Like one respondent commented:

IDI 03: *“I think it’s a very important point with regard to “nothing can be done” and it causes anxiety but let the participant decide. Again where is the line drawn l? If nothing can be done, is that across board? What if I’ve got a cousin say for example in Germany where this technique is available and I can get myself there? Where is the line drawn? We have to be careful because then we’re bringing in inequality of healthcare. Why should a person X not be told because we think they might not be able to act on the results? This might be a burden to them that still brings in that inequality, Humm– particularly -- in terms of monetary management; --- the money aspect also creates inequality—. So my fear is once we start doing that. We do run the risk of creating a very stratified society”*.

In support of the above concept, one respondent said:

IDI 01: *“For some of the interventions it’s not like really if you tell that old woman that you have cancer, who thinks that her kids can’t pay? Who says? It is interesting in this country right now the things that you normally see. Some people can even bring donors on board to help so and so, to go and do this! I have seen some where we have pledged for people that you don’t even know!”*

Another opportunity noted for Botswana’s return of individual results is the national commitment to science, research and innovation. This was seen by some respondents as a way of increasing the quantity and volume of genomic data generated about research participants in Botswana populations. A deeper understanding of this data could arguably benefit research participants when translated into health care interventions. This could translate into a strong argument to afford research participants the opportunity to receive at least some of their individual findings. Due to this need, some of the responses seemed to suggest that there is a need for Botswana to prioritize genomic research since there is a lot that needs to be understood regarding genetic disease and if the results are shared with the participants, it would inform innovations as well as personal value to participants of understanding about their health conditions. Research investigators should take advantage of the infrastructure that government has developed at academic and research institutions which conduct genomic research. Emphasizing this point, one respondent said that:

*IDI 09: “For Botswana there are a lot of opportunities, huge opportunities! They are things that we don’t understand in our nation why they are different from other nations and the hypothesis is that they are driven by host genetics.”*

### Applicable standard of care

The current regulations about the standard of care for research participants in Botswana are yet another opportunity for the return of individual genetic results. For example, the Botswana SOPs and Clinical Trials guidelines expect research investigators and sponsors to provide good standard of care or even better to participants during and after the research as a requirement for demonstrating equal respect for the dignity of research participants especially for multi-site studies. The regulations further elaborate that it is unacceptable for developed-country participants to be offered better standards of care than are offered to Batswana participants in a similar study. In this regard, some respondents felt that this could be a key determinant for and basis for promotion of the return of individual findings as described below:

IDI 10: *“Well, I don’t know if it is very different from what’s happening already. So, that in itself is not completely new and relates back to what we’re feeding back and what is actionable within that setting. I don’t think the government should change the way that they are rationing health care or deciding what they can and can’t afford just based on what we can now test for.*

### Challenges

#### Availability of required resources

Although Botswana has a number of accredited laboratories, some of them might not be able to do genomic validation tests or if they can it is comparatively expensive. Therefore, most respondents expressed concern about the cost of validation of research results especially that of running and maintaining the equipment, acquiring consumables and maintaining expert staff. In addition, the cost of sending samples abroad like the US or, more conveniently, South Africa is also high. Furthermore, lack of genetic health professionals such as medical geneticists and genetic counsellors was considered a problem. For our interviewees, these factors meant that outright promises of returning findings were a challenge as all these bring in a need for financial resources at government and individual level, as well as encourage collaborations to enable access to medical genetic services. Like one respondent said:

IDI 05: “*I mean this confirmation has to be done in an accredited laboratory, which will be expensive. I see these as some of the challenges that confirmations are sometimes done outside the country or even the initial tests. Like here in Botswana, a lot of researchers say we have to send the samples somewhere because we don’t have the capacity and even some of the tests are still being developed. How would you advise government to draft their policy in such a way that we can allow where possible if agreed to collaboration where it [*validation*] can be done outside. You know that processing a material transfer here is a big issue”.*

In the absence of accredited laboratories in the country that can perform genetic sequencing, one of the respondents supported and recommended return of findings which are from standard research methods or sensitive methods that have been used over time by laboratories in the country. However, the respondent emphasized that if the research tools used to test samples are still under validation, results from such tools would need further assessment so should not be fedback to participants. This suggestion was expressed as follows:

*IDI 09: “For me, I think that if for example you are using processes that we call standard methods or standard diagnostic methods or sensitive methods that have been proven over time. I believe such results should be given back to participants. We should be on the benefit side of caution to say that we found a signal that needs to be validated. And then either the signal should be validated or at least there is some information that could be used for further improvement of patient care [----]. Hmm because then people can say Okay, based on this finding we think that it might reach actionable threshold, even though the methodology is not validated. I think the science is improving. Hmm and they are new tools coming up including genomic screening with tools that are still being researched. I think we should make the results available at minimum to the policymakers with further consultation with an Ethics body, medical ethics body, or maybe the participant”.*

Lack of experts in genomics research especially medical geneticists and genetic counsellors was also identified as a big obstacle to the return of individual findings in Botswana. However, considering the small population of Botswana, some respondents thought probably the best solution to this challenge would be to take advantage of the regional and international collaborative partnerships Botswana has established over the years, to attract these experts to build and or strengthen capacity in these areas. One of the respondents supported this suggestion by saying that:

*P 04: Yeah! So the issue of costs is becoming global. For some of the things that involve cost, it is becoming evident that there is no need duplicating. Some of these Technologies can be shared in a cost-effective way. So you find that within in southern Africa, for example, maybe you want in anatomic energies, microscopy or something specialized that costs millions and millions. It could be stationed in Botswana, but everyone around the region can access it”.*

#### Research regulatory oversight

Respondents noted the lack of guidelines specifically for genomic research and expert representation of genomic research experts on IRBs. These shortfalls are critical in guiding the return of individual genetic results. One respondent expressed this concern and proposed a solution as follows:

*IDI 09: “We don’t have systems, governance systems, ethical, legal or policies around these issues and we are waiting until we have an issue to deal with and then we ask ourselves. How do you deal is? But if people would realize that we are now in a global village, yeah, there’s no reason for us not to borrow vessels from elsewhere. Yes, there are International committees, International IRBs; we should be able to network with and say we have this submission. We are interested in getting external assessment”.*

## Discussion

Overall, respondents in our study were of the view that feedback of actionable individual genetic results was an important outcome that could benefit participants. This view is also supported by other studies conducted in response to questions that have been raised in the past decades about the practice of not returning genetic test results and the current push for increased community and participant engagement across the research life-cycle [32, 33]. In this study a number of themes that could impact on return of actionable results surfaced that pointed both to opportunities and challenges to the practice in Botswana.

The question of whether and to what extent genetic research results should be returned to research participants has become one of the most urgent and extensively debated ethical issues in genetics [34]. However, decisions to feedback of findings in genomic research are impacted by, on the one hand a desire to respect participant autonomy by communicating as much information as possible, and a desire to protect participants against harm that may result from sharing poorly validated information [35]. Although the Botswana SOPs [24], section:7.2 (iv) requires that *“participants are informed during the consenting process that the researchers will endeavour to provide information about the outcome of the research, and when it is not intended to provide feedback”*, this requirement is not mandatory and is silent about which information should be disclosed, when and the criteria that determine the return of findings.

According to the current international consensus, results that are fedback must have medical actionability [36]. However, actionability is linked to availability of resources in the genomic research setting that could be highly diverse, especially for multi-country studies or those implemented in regions of a country. Therefore, mapping opportunities and challenges available in a research setting could be a form of assessing the research setting as recommended in the APSRL model as this can impact on medical actionability [6]. This has also been noted by other authors as important since opportunities and challenges may differ between research settings, in terms of available resources to act on the results [37].

The good number of opportunities for the participants in Botswana is encouraging for the research stakeholders to return individual results findings. These opportunities can also offer hope to participants involved in genomic research in Botswana to receive at least some of their results, at least those that are actionable. The most important opportunity identified was the relatively strong health care system that exists in Botswana. This system is supported by good governance, democracy and a culture of humanitarianism, reciprocity and solidarity which cater for availability of most resources necessary for actionability. Strong Health Systems have been found to be lacking in many sub-Saharan countries [38].

Critical among the challenges identified was the strict requirement of feedback of only results validated in an accredited laboratory, a challenge for many sub-Saharan African countries. A survey conducted in forty-nine sub-Saharan countries showed that only 12 of these countries had laboratories that meet international standards. Most of these were located in South Africa (267) while Botswana had six, mostly used for research [39]. To circumvent the problem of diagnostic verification, one respondent proposed that results from robust experimental procedures could provide a useful alternative to facilitate feedback either at the national level or to individuals. National feedback would allow policy makers to plan for validation mechanisms either through establishing collaborative partnerships with settings that have accredited laboratories or develop a quality management system (QMS) for research laboratories testing human biospecimens [6]. With such a system in place, IRBs could permit the return of recommended results under the developed QMS. Alternatively, IRBs could also rely on laboratory analysis that is sufficient to provide confidence in the result, risk benefit analysis and availability of appropriate disclaimer information on the limitations of the validity and interpretation of the individual’s result to permit the return of results. Furthermore, IRBs in Botswana need to develop specific guidelines for determination of return of individual genetic results as has been recommended elsewhere [6, 40].

The cost of subsequent care, for those participants who receive actionable results has been a challenge in many research contexts [41]. From a clinical point of view Botswana has a well-established Universal Health Care system for all its citizens, although variability exists in urban versus rural settings as well as social economic status which can impact on the actionability of findings. The Botswana Integrated Health Services (IHS) system enables linkage of patients to care both regionally and internationally. For example, cancer pathology-based diagnosis and treatment (chemotherapy and systemic surgery) are available at public facilities for free to citizens; radiotherapy is available at some hospitals for free for patients referred through the public facility system [42]. For high-risk variants where treatment is not available through the universal health care system, Botswana’s cultural spirit of self-help, humaneness, solidarity and reciprocity that exist among families and communities compels them to pull together during times of need. This spirit of solidarity has been extended to helping out family and community members seek care from within and outside the country through fundraising which gives hope to participants.

Respondents also raised concern about lack of genomic clinical expertise to confirm data quality, perform variant assessment to determine significance of results, and effectively communicate results to participants as has been mentioned elsewhere [43]. Lack of genetic counsellors was also viewed as critical by some respondents who felt that counselling was important for the delivery of comprehensive genomic medicine. The roles of genetic counsellors required for interpretation, explanation and feedback of genetic results, support of participants and their families in decision-making, calculation and prediction of risks of genetic disease and handling all the consequent psychosocial and ethical issues that may arise require specialized training [44]. Unavailability of guidelines specific to requirements for determination of return of individual genetic results as well as lack of genomic research expert representation on IRBs were also noted as a barrier to ethical review decision-making. Since in Botswana, genomic research is still in its infancy, there is need for capacity building in this area of expertise and it is hoped that with the country’s commitment to developing capacity in genomic research, this problem could be solved in the near future. Also noted as a constraint was the lack of IRBs having representatives who are specialized in genomics on the local ethics committees. It was considered important for Botswana to set up Ad hoc committees with members specialized in genomics to review proposals submitted in this field. Other solutions to the challenges faced by local IRBs included the possibility of inviting expert genomics external reviewers from within or outside the country to review genomic research proposals, develop guidelines specific for appropriate and clear guidance of return of individual genetic results and at the same time develop local IRB capacity in genomic research through training.

### Study limitations

Genomic research in Botswana is in its infancy and the study was conducted in the capital city Gaborone with only a few research and academic institutions involved in genomic research; thus the small sample size. However most of the respondents had long experience in clinical and socio-behavioural research while others were long serving members of IRBs and Community Advisory Boards. Secondly a number of respondents were both health care providers and researchers which could have introduced some bias to the responses provided.

## Conclusion

We describe opportunities and challenges for the return of individual genetic results in Botswana regarding the availability of the necessary resources for actionability. Generalizing availability of resources across different settings could lead researchers to make false assumptions about what is (not) actionable. If actionability is one of the key criteria influencing decisions on what to feedback, then equally as such, the return of results should be proximally focused to the opportunities and challenges that exit in a setting where the results emerge in order to guide actionability. Furthermore, as the contexts and policies for return of results continue to evolve; this will continue to create gaps between availability of resources to meet these requirements of those who have and those without, threatening international collaboration. Therefore, we propose that decisions whether and which genetic results to return take into consideration prior mapping of contextual opportunities and challenges for actionability requirements for return of results in a research setting. This is likely to avoid or minimize ethical issues of justice, equity and harm regarding actionable decisions.

## Electronic supplementary material

Below is the link to the electronic supplementary material.

Additional File 1: In-depth Interview Questionnaire

## Data Availability

The datasets used and/or analysed during the current study are available from the corresponding author on reasonable request using the email address provided.

## Abbreviations

APRSL: Actionability at the Participant Research Setting Level
CABs: Community Advisory Boards
CAfGEN: Collaborative African Genomics Network
GDP: Gross Domestic Product
H3Africa: Human, Health and Heredity in Africa
*HIV*: Human immunodeficiency virus
IRB: Institutional Review Boards
QMS: quality management system
SOPs: Standard Operating procedures
TB: Tuberculosis
VDC: Village Development Committees
