# Supplementary material for: Feeding back of individual genetic results in Botswana: mapping opportunities and challenges
Source: BMC Med Ethics. 2023 Jun 3;24:37. doi: 10.1186/s12910-023-00912-1 (PMC10239568; doi:10.1186/s12910-023-00912-1)
Supplement: Supplementary file 1 — Additional File 1: In-depth Interview Questionnaire [file 12910_2023_912_MOESM1_ESM.docx]

**PROJECT TITLE: Prospective analysis of stakeholder expectations and preferences for Feedback of Individual Genetic Findings in genomics research in Botswana**

**Principal Investigator(s): Prof. Mogomotsi Matshaba and Dr. Mary Kasule**

**South Africa Co-Investigators: Prof. Ambrose Wonkman & Prof. Jantina de Vries,**

**Co-Investigators: Mrs Keofentse Mathuba. Dr. Abhilash, Ms. Dimpho Ralefala**

**Study Contact telephone number**: +267 71862559/3552911

**Protocol Version: Version 1 dated 23/02/2018**

**UNIVERSITY OF BOTSWANA**

**STAKEHOLDERS** **– IDIs with people involved in the development or implementation of returns policies in Botswana**

What we want to get from the interviews is a sense of what people involved in the development, review or implementation of returns policies think about the return of individual genetic research findings for genomics research. Specifically, we will speak with:

- Genomic researchers and genetic healthcare professionals involved in research;
- Members of ethics committees
- National and local policy makers
- Members of the Rare Disease Communities in SA and Botswana, including e.g. representatives of patient advocacy groups and healthcare professionals

Questions/purpose of FGDs:

1. Explore what interviewees know about genomics and the data it generates
2. Explore what people know about how genomic information relates to individual health
3. Explore whether interviewees think (some) results should be returned, why and how
4. Explore what the implications would be of such a policy and who should bear the cost

**Introduction**

Thank you for your willingness to participate in this interview and contributing to our project. My name is Dr. Mary Kasule from the University of Botswana. Our research team is working on a project known as the **Individual Findings in Genetics Research in Africa (IFGeneRA) H3Africa**. The team aims to progressively build an evidence base that forms the basis for context- and country specific development of policies relating to the returns of individual genetic research results for African genomic research. A key ethical challenge in genomics research relates to whether and which individual genetic research results ought to be fed back to research participants. Although a lot has been published on genomics research in recent years, the feedback of results has received virtually no attention from African scholars. These interviews will be conducted both here in Botswana and South Africa. The interview will last approximately 45 minutes to one hour. We would like to record and transcribe this conversation in order to supplement our notes and accurately share our findings. Your information will be kept confidential and we will **NOT** quote your words or connect your name with what you say in any of our reports unless you give us permission. Interview transcripts will only be shared with the research team. You do not have answer all the questions but if there is anything you think the research team should know regarding this project, you are welcome to share your thoughts with the team. Before we start, I would like to know if you have any questions.

**Do I have your permission to record this consultation? YES/NO**

- Introduce self, organisation
- Introduce study
- Why am I interviewing you?
- Length of Interview
- This interview part of a study that will conducted both in Botswana and South Africa and Cameroon
- Explain why recording
- Confidentiality reminder – *interview transcripts only shared with research team*
- Voluntariness reminder – *no need to answer all questions*
- Compensation and potential harms.
- Any questions?
- Consent form and signing of the form
- Should I write this in detail as an introduction)

**Topic 1 Background**

I will first ask you some questions about your background, and about what you know about genomics.

1. Explore professional background, current job and length of time in that job
2. Knowledge of genomics
   1. How have you encountered genomics in general and research in your work?
   2. What do you know about genomics?
   3. Do you remember any particular examples of genomic studies that were conducted?
3. What do you think we can find out about research participants on the basis of their genomic information?
4. Specifically, how do you think genomic data could be relevant to the health of a person? *Use this section of the interview to develop a good idea of the participants’ understanding of genomics. Don’t move on to the next section until you’ve developed such an idea.*

**Topic 2 Feedback of results to participants**

In this study, we are interested to know what kind of individual genetic research results should be fed back to research participants, if any. In the next section, I will ask you some questions about this

In the next section, I will ask you some questions about this.

1. In **any** research, do you think any individual research results should be fed back? Which ones and why?
   - E.g. blood pressure information during recruitment
2. Why?
   - Health benefits from actionable information
   - Respect for autonomy
   - Engaging participants in the research process/empowering
3. Why not?
   - Research intended to provide generalizable knowledge
   - Provision of results can be confusing (therapeutic misconception)/harmful/diverting resources from research to providing results could be inappropriate
4. If a researcher finds that a participant has high blood pressure, what do you think they should do with that information?
   - Tell the participant?
   - Pay for the participant to see another doctor?
5. What kind of information should not be fed back?
   - Information that is harmful? (Examples?)
   - E.g. false paternity?

**Which genomics research what kind of individual genetic research results should be fed back?**

Genomics research can identify findings relevant to the health of individuals by providing information on every mutation that influences risk of disease. The Whole Genome sequencing (WGS) tool can assess virtually every gene in the human genome for disease-predisposing variants. Sometimes there are multiple, and potentially hundreds, of individual genetic research results (**secondary or incidental results)** that can be offered for return to individuals and families. However, the lack of clear guidance about whether and, if so, how, when and who should return the results limits the extent to which individuals and families are empowered to translate genomic information into improved lifestyles, medical care, and ultimately long-term health. In the US and Europe, scientists have agreed that genetic information relating to different kinds of cancers and heart disease could be considered for feedback.

Do you think researchers should feedback individual genetic research results? Why?

- Considerations: obligation differences between researcher and clinical care providers; aims of research and clinical care (therapeutic misconception); right to know; analytical validity of results;

1. Which kinds of results should be fed back?
   - Those relevant to health?
     - All relevant health results?
     - Only for very severe, possibly lethal conditions?
   - Those relevant to a person’s ancestry?
   - Other kinds of results?
2. Why? Could it because
   - Its theirs
   - there are potential benefits (diagnosis, prevention and treatment)
   - supporting autonomy
   - Empowerment
   - reciprocity
   - Improved trust between researchers and society
3. When genomic research enrols children, should the parents receive results about their children?
   - What about late onset conditions, e.g. breast cancer?
   - Do parents have a right to know everything about their children? What about the children’s autonomy (especially when growing up)
4. Sometimes, researchers may want to feedback results not because it would help the participant but because it could help their family members. For instance, this would be the case if they find that one of their male participants carries variants relating to breast cancer – knowing that wouldn’t help them, but it would help their sisters and daughters. In that case, do you think those results should be fed back?
5. How do you think the participants would like to receive the results?
   - Report sent through post or email
   - A phone call from a genetic counsellor
   - A referral doctor
   - Face-to-face meeting with a doctor or genetic counsellor
6. When should the results be fed back?

**Actionability**

Internationally, the consensus is that only results that are ‘actionable’ should be fed back. This means that some kind of intervention is available that participants can take to prevent the illness from developing. This could be something concrete – for instance, an operation to insert a device in the heart, or removing the breasts so that women don’t develop cancer. It could also be something softer – for instance, regular screening, or changes in diet and exercise.

1. Do you agree with this criterion of ‘actionability’? Is it right that only results that the person can do something about should be fed back?
   - E.g. Alzheimer’s or Huntington’s disease – ok not to feedback?
2. How do we know that something is actionable for each individual person though?
   - E.g. woman in a remote rural area may not have access to operation – still ok to feedback?
3. If something is actionable for some participants in a project, but not for others (e.g. in a different country) would it be ok to feedback information only to some people but not to others?

**Cost of follow-up care**

Even when researchers give back individual research results, the patients would still need to see a doctor to go for confirmation testing. They may also need an operation (for instance, if there is a risk of developing cardiomyopathy or breast cancer) or they need annual screening. That means there is a cost associated with knowing you have a genetic predisposition. It also means that the information is not definite but indicates a risk. Considering that there is a cost associated with receiving genetic research information, do you still think this information should be fed back? Why?

1. What would be the cost of integrating WGS into Primary Care?
   - **(**Based on the data collected from NHGRI-funded **genome**-**sequencing** groups, the **cost** to generate a high-quality 'draft' whole human **genome sequence** in mid-2015 was just above $4,000; by late in 2015, that figure had fallen below $1,500. The **cost** to generate a whole-exome **sequence** was generally below $1,000).
   - Aabsence of services of genetic counselors and clinicians
   - resources to use the services of laboratories that are accredited to verify the accuracy of the results,
   - Legal requirements
   - IRB authority
   - CAB authority
   - Policy makers
2. Who should pay for follow-up care? Why?
   - Explore the responsibilities of researchers, government, research participants
   - How far do the responsibilities of the researchers extend? E.g. should they only arrange a clinical appointment, should they pay for the consultation etc?
   - Only be responsible for mutations that fall into the research teams’ area of expertise?
   - Remember that the real interest here is understanding why, with a particular focus on reciprocity.

**Taking a second sample (Confirmation):**

When doing research, it is always possible that some of the tubes get mixed up, or that the results for different people get mixed up. For research, that is not a problem (the overall dataset is still the same) but it would be very bad for individuals to get the wrong results of course. Some people have proposed that the solution is to approach participants and ask them for a second sample, but this has implications for cost and logistics. Regulations under the Clinical Laboratory Improvement Amendments of 1988 (CLIA) define requirements for clinical laboratories intended to ensure that laboratory tests used in clinical care meet acceptable quality standards. Laboratories that supply test results for use in clinical care must be CLIA-certified or CLIA exempt (together, CLIA-compliant).

1. Do you think researchers should ask participants for a second sample? How do they ask for this?
   - E.g. ‘we may have found something but we want to know for sure’ – wouldn’t that make people suspicious or worried?
2. Re-contacting people, making appointments, taking another sample, screening the other sample – this is expensive. Who should bear the cost of this?
   - Researchers, funders, the healthcare system?
   - Availability of organizational and human resources (genetic counsellors)
   - IRB and community engagement
3. If the trade-off is between not giving results or giving results with the warning that there may have been mix-up (but without collecting a second sample), do you still think researchers should feedback individual results?

**Consent**

Most studies do not seek consent for feedback of results, partly because genomics studies are already difficult to explain.

1. Do you think researchers should feedback individual genetic research results if they did not ask for consent to do so? Why?
2. Do you think going forward that participants should be asked for consent? For general feedback, individual feedback?
   - But what if researchers find something that signal predisposition to something quite lethal (e.g. breast cancer in women) and people said ‘no I don’t want to know’. Have they really considered all the issues?

**Implications for research**

When analysing their study data, researchers would not normally look for mutations that are associated with diseases. If the decision is to feedback certain study results, then the team would have to go out and look for these mutations. This takes time and resources.

1. Do you think it is appropriate that researchers spend time and resources to look for relevant personal mutations?

**Topic 3 Closing Section**

- We have now reached the end of this interview. Is there anything else you think is important that we have not talked about today?
- Do you have any questions about what we talked about? Is there anything you need more information about or you would like to share with the team?

***Thank you very much for participation in this study.***
